# Dr. William Herbst

**Published:** 1886-09

**Authors:** 


DR. WILLIAM HERBST.—The reception extended Dr. William Herbst during his recent visit to this country has been of the most cordial character, and he will no doubt have been impressed with the fact that true merit is always recognized by those of our profession whose recognition and appreciation are worth having. The following is an account of a clinic held at the office of Dr. Bodecker, of New York, and which is described in the *Independent Practitioner:*

" Dr. J. C. Proud took the operating chair, and Dr. Herbst filled for him the right upper second bicuspid, the cavity occupying the distal and grinding surface of the tooth. Previous to the excavation a matrix of German silver was prepared in the following manner: A piece of German silver, No. 32 American guage, one-quarter of an inch in width and one and one-half inches in length, was passed around the bicuspid to be filled. Then, by means of a blunt pair of cutting forceps, which in form somewhat resemble a pair of flat-nosed pliers, it was drawn around the tooth in such a way as to make it fit very closely. The German silver ring was then withdrawn from the tooth, a little soldering fluid (solution of chloride of zinc) was applied to the flanges and the ring soldered. One of the principal points to be remembered in the making of this matrix is to avoid allowing the tin solder to run to the side of the matrix next the cavity to be fllled, as when the tin is touched by the rotating instrument some of it will be incorporated into the gold and the cohesion of the separate layers of gold impaired. Previous to soldering the matrix must be made perfectly clean, and this is done by means of a piece of cotton wound around an engine bur dipped into moistened pumice stone, and this rubbed over the ring, inside as well as outside. After the cavity had been thoroughly excavated, the rubber dam and the matrix were applied and the filling introduced upon the cervical wall of the cavity. Dr. Herbst placed a very thin layer of tin, burnishing it up over the pulp, which was very nearly exposed, thereby modifying thermal changes. The tin was then followed by a layer of Wolrab's gold cylinders, No. 0, condensed in the usual way, first with the hand instrument and then with an agate point in the dental engine. When this was completed, a fine instrument made of a broken excavator was pressed all over the surface, first to discover all imperfectly condensed plates, and secondly to roughen the polished surfaces of the gold, thereby obtaining better unity of the layers of the filling. The introduction of the gold occupied about eighteen minutes. The gentlemen present at the meeting were Drs. Taft, Bonwill, Abbott, McKellops, Rehwinkel, Tennison, Andrews, V. Pressler and Rhein.

In the afternoon another clinic was given, at which Dr. Herbst filled a lower molar handed him by Dr. Dwinelle, which had been extracted over thirteen years. the cavity occupying the mesial and grinding surface of the tooth. Previous to excavating the cavity, a German silver matrix was applied, as mentioned before, but in addition to the matrix a brass wire was soldered all around it with soft solder, in order to stiffen it. After the cavity had been excavated, the tooth was imbedded, with the matrix in position, in a large piece of shellac. The first layers of gold were condensed by means of a piece of cotton in the following manner: Cotton enough to fill the cavity was put over the gold, and then, by means of a large smooth engine burnisher, it was burnished into every corner where there was any gold; when the cotton was removed, it showed that the gold was thoroughly depressed into every portion of the cavity, and it was then further condensed by means of agate instruments in the dental engine. Dr. Herbst here remarked that the cotton, when applied in this manner, will make the most cohesive gold non-cohesive, but the moment rotating instruments have been used upon the surface it will again become as cohesive as before. The rest of the layers of gold were applied in the usual way, until the cavity was nearly full, when one of the walls of the tooth gave way on account of the desiccated condition of the dry tooth, and the defect had to be repaired with gold, without removing the matrix, although the edges of the tooth could not be made perfectly smooth. When it was filled, the shellac and the matrix were removed, and the gold was found to be absolutely perfect in every respect. The filling was then finished with sandpaper disks, without burnishing the outer surface, so as to see whether the gold had reached all the rough edges of the broken wall. It was very critically examined by Drs. Taft, Dwinelle, Rehwinkel, Parr, Northrop, and Bogue, all of whom agreed that the operation was absolutely perfect.

Dr. Herbst then filled a cavity in a steel matrix (the same one used by Dr. E. Parmley Brown for ascertaining the comparative weight of gold when condensed by the different methods of filling teeth), into which he introduced Wolrab's gold cylinders, No. 0 by means of hand instruments, assisted by his new agate points. This matrix was filled in about nineteen minutes, and the filling, when completed, weighed eighteen and one-sixteenth grains, or, one-sixteenth of a grain more than the filling introduced by Dr. Brown, with the electric mallet and an extra powerful battery, in forty minutes time.

Dr. Dwinelle to satisfy himself as to the possibility of condensing gold by means of a piece of cotton, took an engraved stone, and after he had introduced a few large Wolrab cylinders, applied a piece of cotton over it and condensed the gold with it. When removed from the matrix, the gold was found to be a beautiful and exact counterpart of the engraved stone."

